# Value of virtual reality integrated with multilevel team-based pedagogy in standardized residency training: a randomized controlled study with longitudinal follow-up in anesthesiology

**DOI:** 10.3389/fmed.2026.1745346

**Published:** 2026-02-03

**Authors:** Si Chen, Chuanguang Wang

**Affiliations:** Department of Anesthesia, Lishui Municipal Central Hospital, The Fifth Affiliated Hospital of Wenzhou Medical University, Lishui, Zhejiang, China

**Keywords:** anesthesiology education, clinical competence, randomized controlled trial, team-based learning, virtual reality

## Abstract

**Background:**

Achieving both technical and non-technical competencies in anesthesiology residency training remains challenging, highlighting the need for innovative educational strategies.

**Methods:**

In this single-center randomized controlled trial, 120 anesthesiology residents were assigned 1:1 to virtual reality (VR)–integrated team-based pedagogy (VR-TBP, *n* = 60) or conventional training (*n* = 60). The intervention combined immersive VR simulations with multilevel team-based teaching, while controls received standard lectures and bedside instruction. Primary outcomes included first-pass tracheal intubation success, intubation time, procedural errors, and ultrasound-guided nerve block performance. Secondary outcomes were Mini Clinical Evaluation Exercise (Mini-CEX), Anesthetists’ Non-Technical Skills (ANTS), theoretical knowledge, self-efficacy, and satisfaction. Long-term endpoints at 6 and 12 months assessed skill retention, independent procedure completion, and adverse events.

**Results:**

Baseline characteristics were comparable. At 12 months, VR-TBP participants achieved higher first-pass intubation success (86.7% vs. 68.3%, *p* = 0.026), shorter intubation times (60.1 ± 11.0 vs. 66.8 ± 12.6 s, *p* = 0.006), fewer errors (1.4 ± 0.7 vs. 2.0 ± 0.9, *p* = 0.007), and greater nerve block success (81.7% vs. 65.0%, *p* = 0.041). Non-technical outcomes also favored VR-TBP, with higher Mini-CEX (6.7 ± 1.0 vs. 5.9 ± 1.1, *p* < 0.001) and ANTS scores (11.5 ± 1.6 vs. 9.9 ± 1.7, *p* < 0.001). Skill retention (88.4% vs. 76.5%, *p* < 0.001) and independent procedure completion (76.7% vs. 58.3%, *p* = 0.032) were superior, with comparable adverse event rates.

**Conclusion:**

Integrating VR-based simulation with team-based pedagogy significantly enhanced technical and non-technical competencies among anesthesiology residents, with sustained benefits at 12 months. VR-TBP offers an effective, reproducible model to strengthen residency training.

## 1 Introduction

High-quality anesthetic care depends on the competence of residents who must acquire both technical and non-technical skills to ensure perioperative safety (1). Core technical abilities—such as tracheal intubation, ultrasound-guided regional anesthesia, and emergency crisis management—must be complemented by non-technical domains including teamwork, communication, task management, and situational awareness (2). Together, these competencies determine not only procedural success but also patient outcomes in complex clinical environments. However, conventional residency programs remain dominated by lectures and bedside teaching, which provide limited opportunities for structured, high-fidelity practice and collaborative learning (3). These constraints have raised increasing concern regarding whether traditional training approaches adequately equip residents for the demands of modern anesthetic practice (4).

Simulation-based education has emerged as a valuable adjunct to clinical training by offering standardized, reproducible, and risk-free environments for deliberate practice (5). Among these modalities, virtual reality (VR) is distinguished by its ability to create immersive and interactive scenarios that mimic real-world complexity (6). VR has demonstrated promising results in selected domains such as airway management and perioperative crisis simulation, enhancing procedural accuracy and accelerating skill acquisition (7). Nevertheless, prior investigations have typically been restricted by small sample sizes, limited training duration, or a focus on isolated technical skills. Moreover, the durability of VR-related benefits and its integration into structured pedagogical frameworks remain insufficiently evaluated (8).

In parallel, educational theory highlights the importance of team-based pedagogy, in which residents of varying seniority assume collaborative roles and engage in structured feedback. Such multilevel interactions not only reinforce technical precision but also cultivate decision-making and communication under pressure—skills essential for real-world anesthesiology practice. Despite these theoretical advantages, few randomized controlled trials have rigorously examined whether combining VR with structured team-based pedagogy (VR-TBP) can produce measurable and sustained improvements in resident competence (9).

Against this background, the present randomized controlled trial was designed to evaluate VR-TBP compared with conventional training in anesthesiology residency. By enrolling 120 participants and performing assessments at baseline, immediately after training, and at 6- and 12-month follow-up, the study aimed to provide robust evidence on both short-term gains and long-term retention (10). Outcomes encompassed technical proficiency, non-technical performance, knowledge acquisition, and clinical independence (11). This design addresses critical gaps in the literature and offers novel insights into whether VR-TBP can serve as an innovative, scalable framework for modernizing residency training in anesthesiology (12).

## 2 Materials and methods

### 2.1 Study design and participants

This investigation was conceived as a prospective, randomized controlled trial conducted at Lishui Municipal Central Hospital in China, a designated training center for standardized anesthesiology residency. The trial evaluated the impact of integrating VR–based simulation with a team-oriented pedagogical framework compared with conventional instruction. The study adhered to the CONSORT 2010 guidelines, with standardized assessments at three fixed time points: immediate post-intervention (T1), 6-month follow-up (T2), and 12-month follow-up (T3). The protocol was approved by the institutional ethics committee, and written informed consent was obtained from all participants.

Eligible residents were those in the first to third year of anesthesiology residency (R1–R3), aged 24–30 years, who had completed prerequisite theoretical examinations permitting progression to clinical practice.

### 2.2 Randomization and interventions

Participants were randomized in a 1:1 ratio using a computer-generated sequence, with allocation concealment via sequentially numbered, opaque, sealed envelopes prepared by an independent coordinator. Outcome assessors for the Mini Clinical Evaluation Exercise (Mini-CEX), Objective Structured Assessment of Technical Skills (OSATS), Anesthetists’ Non-Technical Skills (ANTS), and General Self-Efficacy Scale (GSE) were blinded to group assignments.

Residents in the VR–TBP group completed a 6-week intervention consisting of one 60-min orientation session (Week 0) and six 90-min VR training sessions (Weeks 1–6), scheduled once weekly (total planned dose: 11 h per participant). Training was delivered using a head-mounted display (HMD) VR system with dedicated anesthesia modules. Team roles (operator, assistant, observer, and recorder) were rotated within each session, and every session ended with a structured debriefing. The VR platform automatically recorded objective performance metrics (e.g., completion time, critical errors, and scenario-specific success measures) that were reviewed during debriefing. After completion of the 6-week intervention, no further VR sessions were provided. Specifically, there was no VR exposure between T1 and T2 (6-month follow-up) or between T2 and T3 (12-month follow-up). During follow-up, both groups continued routine clinical rotations and standard residency teaching; access to the VR system was restricted to minimize contamination. The VR training was delivered using a standalone head-mounted display (HMD) system (PICO Technology Co., Ltd., PICO 4; Beijing, China) with inside-out 6-degree-of-freedom (6DoF) head and controller tracking. All scenarios ran on a dedicated anesthesia VR education application (custom-developed for residency training) that provides interactive modules for airway management, ultrasound-guided peripheral nerve blocks, and intraoperative crisis management. The same hardware and software installation package was used for all participants throughout the study, and automatic updates were disabled to ensure a consistent training environment.

The control group received conventional training during the same 6-week period, including lectures, bedside teaching, and routine skills practice aligned with the same topic areas and assessment schedule. Total contact time (time-on-task) was matched to the intervention group; the key difference was the absence of any VR component.

### 2.3 Outcomes and assessments

Primary endpoints were technical performance: first-pass tracheal intubation success, intubation time, procedural errors, and ultrasound-guided nerve block success. These were assessed at T1, T2, and T3 using standardized checklists validated by senior faculty.

Secondary outcomes included Mini-CEX, ANTS (task management, teamwork, situational awareness, decision-making), theoretical knowledge, self-efficacy (GSE), and training satisfaction ( ≥ 4/5 on a 5-point Likert scale). OSATS was assessed at baseline for comparability.

Long-term outcomes at 6 and 12 months included: (i) skill retention (percentage of T1 technical performance maintained at follow-up), (ii) proportion of residents achieving independent procedural performance, and (iii) adverse event incidence. Long-term endpoints included skill retention, proportion of residents independently completing designated procedures, and adverse events. All tools employed validated Chinese versions; where full psychometric validation was unavailable, internal consistency and inter-rater reliability were assessed within this study.

### 2.4 Sample size

Sample size was estimated using PASS 15.0 software (NCSS, LLC, Kaysville, Utah, United States), with the practical assessment score as the primary outcome. Preliminary data (13) from our pilot study showed scores of 6.47 ± 1.45 in the conventional teaching group and 7.73 ± 1.22 in the VR group (mean ± SD), corresponding to an expected mean difference of 1.26 points. Assuming a two-sided α of 0.05, a power (1−β) of 0.80, equal group sizes, and a two-sample *t*-test with equal variances, PASS indicated that 19 participants per group (38 in total) would be required. Allowing for an anticipated attrition or missing-assessment rate of approximately 20%, the target sample size was inflated to 24 participants per group, for a total of 48 participants. On this basis, the planned sample size was considered adequate to detect a meaningful difference in the primary outcome. In addition, a post hoc power analysis was performed, which yielded a statistical power of 0.95. This result indicates that, with the final sample size, the study had more than adequate statistical power to detect differences in the primary outcome, further confirming the robustness of the sample size determination.

### 2.5 Statistical analysis

Statistical analyses were performed in SPSS 25.0 and R 4.3.1. Continuous variables are reported as mean (SD) or median (IQR), and categorical variables as n (%). Baseline between-group comparisons used *t*-tests or Mann–Whitney U tests, and χ^2^ tests or Fisher’s exact tests, as appropriate. Longitudinal outcomes (T1–T3) were analyzed using prespecified repeated-measures models to account for within-participant correlation. Continuous outcomes were analyzed with linear mixed-effects models including fixed effects for group, time (categorical), and group × time, and a participant-level random intercept. Binary outcomes were analyzed with generalized estimating equations (logit link; exchangeable working correlation) including the same fixed effects. We report the group × time interaction and time-specific marginal contrasts (VR–TBP vs. control) with 95% CIs. Pairwise comparisons were adjusted using Bonferroni correction where applicable. Effect sizes are presented as mean differences or odds ratios/risk ratios with 95% CIs. Two-sided *p* < 0.05 was considered significant. Analyses followed the intention-to-treat principle. Missing data were assumed missing at random; primary models used all available observations, with multiple imputation as a sensitivity analysis (combined using Rubin’s rules). Details of missingness and imputation are reported in the Results.

## 3 Results

### 3.1 Baseline characteristics of participants

**FIGURE 1:**
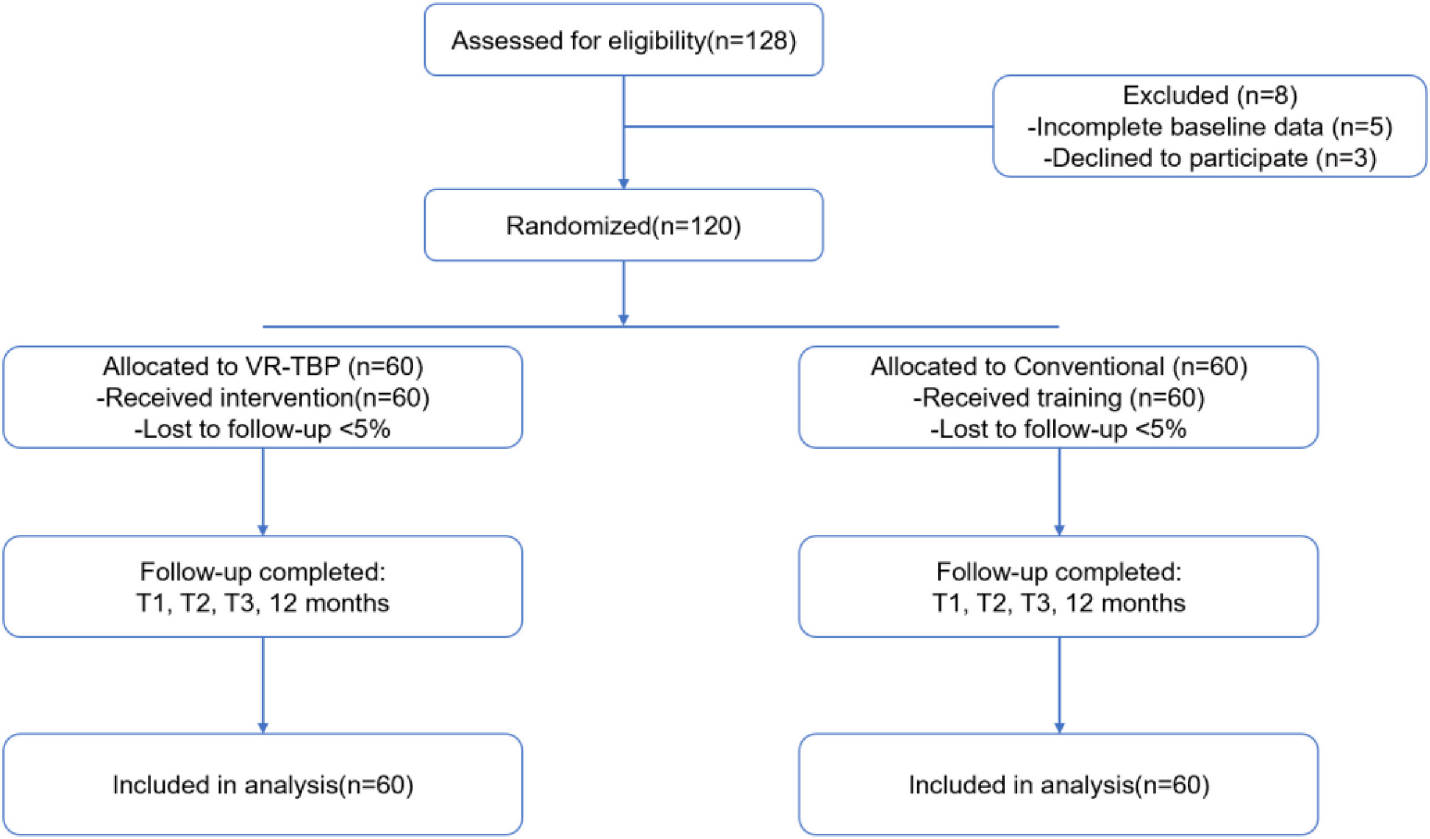
Flowchart. A total of 128 anesthesiology residents were assessed for eligibility. Eight residents were excluded due to incomplete baseline data (*n* = 5) or declining participation (*n* = 3). The remaining 120 residents were randomized equally to the intervention group (VR-TBP, *n* = 60) or the control group (Conventional, *n* = 60). All allocated participants received the assigned training. Follow-up assessments were completed at immediate post-intervention (T1), 6 months (T2), and 12 months (T3). Attrition during follow-up was < 5% in both groups, and 120 residents were included in the final analysis.

Of 128 anesthesiology residents screened, 120 were enrolled and randomized equally to VR-TBP (*n* = 60) or conventional training (*n* = 60), with < 5% attrition during follow-up (Figure 1). Baseline demographic and training characteristics were well balanced between groups (Table 1). The mean age was 28.1 ± 2.7 years in VR-TBP and 27.8 ± 2.5 years in controls (*p* = 0.57), with similar sex distribution (50.0% vs. 46.7%, *p* = 0.79), residency year, and prior intubation experience. Training duration was also comparable (11.7 ± 3.2 vs. 11.4 ± 3.0 months, *p* = 0.68).

**TABLE 1:** Baseline characteristics (*N* = 120).

Characteristic	Control group (Conventional, *n* = 60)	Intervention group (VR-TBP, *n* = 60)	Statistical test (value)	*P*-value
Age, years, mean (SD)	27.8 (2.5)	28.1 (2.7)	Independent *t*-test (*t* = −0.57)	0.57
Sex, male, n (%)	28 (46.7)	30 (50.0)	Chi-square (χ^2^ = 0.07)	0.79
Residency year, n (%)	R1: 20 (33.3); R2: 20 (33.3); R3: 20 (33.3)	R1: 18 (30.0); R2: 21 (35.0); R3: 21 (35.0)	Chi-square (χ^2^ = 0.20)	0.90
Training duration, months, mean (SD)	11.4 (3.0)	11.7 (3.2)	Mann–Whitney U (*Z* = −0.42)	0.68
Prior independent intubation experience (≥5 cases), n (%)	15 (25.0)	17 (28.3)	Chi-square (χ^2^ = 0.16)	0.69
Baseline theoretical exam, score (0–100), mean (SD)	72.4 (5.7)	73.2 (6.1)	Independent *t*-test (*t* = −0.70)	0.49
Mini-CEX global score (1–9), mean (SD)	5.9 (1.0)	6.0 (1.1)	Independent *t*-test (*t* = −0.41)	0.68
OSATS technical skills (0–25), mean (SD)	13.4 (2.7)	13.6 (2.8)	Independent *t*-test (*t* = −0.37)	0.71
ANTS non-technical skills (4–16), mean (SD)	9.5 (1.5)	9.6 (1.6)	Independent *t*-test (*t* = −0.34)	0.74
General Self-Efficacy Scale (10–40), mean (SD)	28.0 (3.5)	28.4 (3.6)	Independent *t*-test (*t* = −0.59)	0.56
Training satisfaction (Likert ≥ 4/5), n (%)	44 (73.3)	45 (75.0)	Chi-square (χ^2^ = 0.04)	0.85

Academic and specialty-related indicators likewise showed no significant differences (Table 1). Theoretical exam scores (73.2 ± 6.1 vs. 72.4 ± 5.7, *p* = 0.49), Mini-CEX (6.0 ± 1.1 vs. 5.9 ± 1.0, *p* = 0.68), OSATS (13.6 ± 2.8 vs. 13.4 ± 2.7, *p* = 0.71), ANTS (9.6 ± 1.6 vs. 9.5 ± 1.5, *p* = 0.74), and self-efficacy (28.4 ± 3.6 vs. 28.0 ± 3.5, *p* = 0.56) were all comparable. Training satisfaction was high in both groups (75.0% vs. 73.3%, *p* = 0.85).

These findings confirm that the two groups were well matched at baseline across demographic, academic, and training-related characteristics.

### 3.2 Technical skill outcomes

**FIGURE 2:**
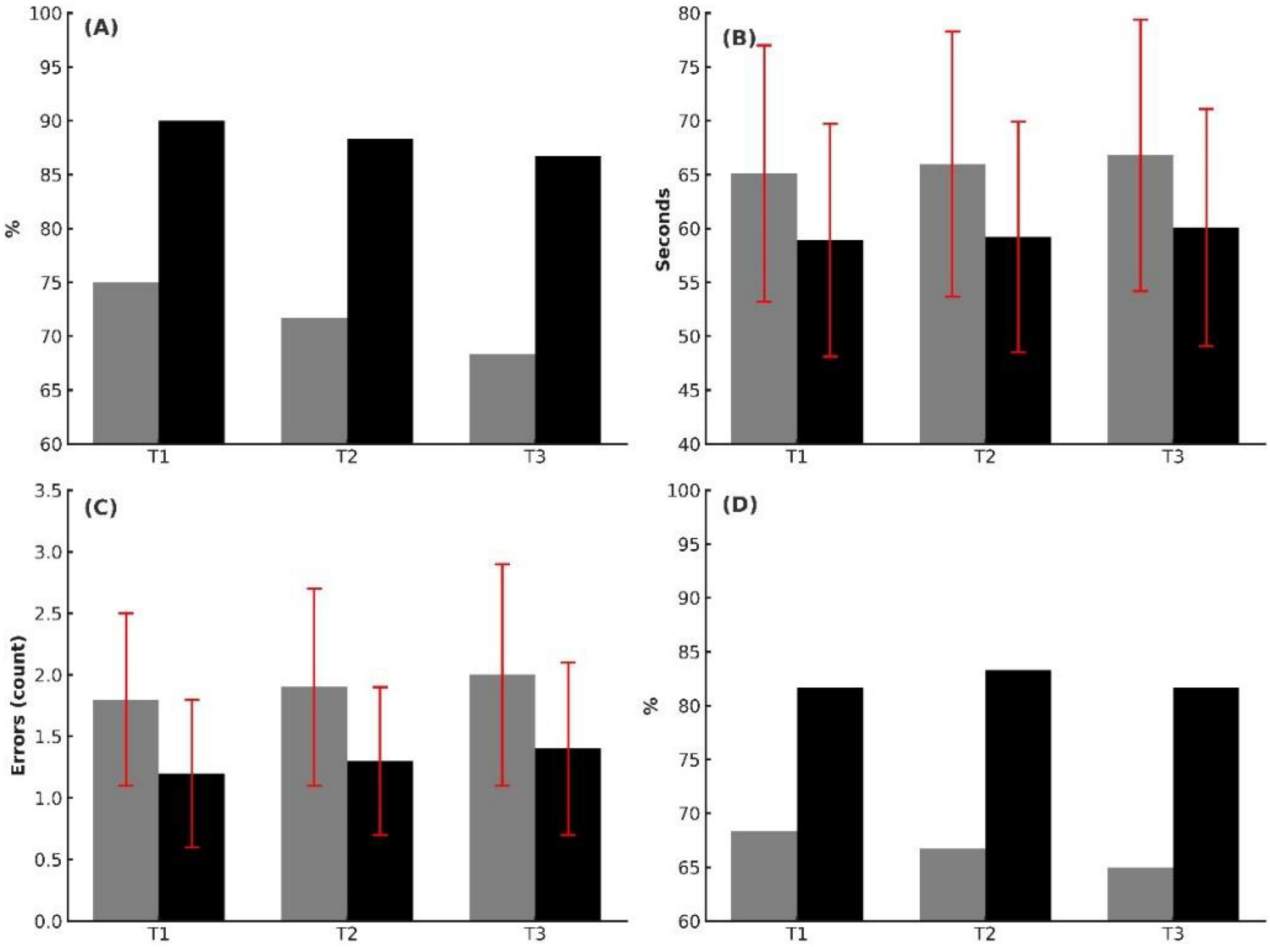
Technical skills outcomes at T1 (immediate post-intervention), T2 (6-month follow-up), and T3 (12-month follow-up). **(A)** Intubation success rate (%), showing consistently higher rates in the VR-TBP group compared with Control. **(B)** Intubation time (seconds, mean ± SD), with significantly shorter times in the VR-TBP group across all time points. **(C)** Procedural errors per intubation (mean ± SD), demonstrating fewer errors in the VR-TBP group. **(D)** Nerve block success rate (%), with higher success in the VR-TBP group, reaching statistical significance at T2 and T3.

Longitudinal assessment demonstrated consistently better technical performance in the VR-TBP group compared with conventional training (Figure 2; Table 2). First-pass intubation success was higher in VR-TBP residents at all-time points, with significant differences maintained through 12 months (T1: 90.0% vs. 75.0%, *p* = 0.038; T2: 88.3% vs. 71.7%, *p* = 0.025; T3: 86.7% vs. 68.3%, *p* = 0.026). Intubation times were consistently shorter (e.g., T3: 60.1 ± 11.0 vs. 66.8 ± 12.6 s, *p* = 0.006), and procedural errors fewer (T3: 1.4 ± 0.7 vs. 2.0 ± 0.9, *p* = 0.007).

**TABLE 2:** Primary technical outcomes and OSATS scores of anesthesiology residents at immediate post-intervention (T1), 6-month follow-up (T2), and 12-month follow-up (T3).

Indicator	Time point	Control (*n* = 60)	VR–TBP (*n* = 60)	Effect at time point (VR–TBP vs. control)	Group × time interaction, test statistic (df)	*p*-value (interaction)
Intubation success rate	T1	45 (75.0%)	54 (90.0%)	OR 3.00 (1.08–8.37)	Wald χ^2^(2) = 0.30	0.861
	T2	43 (71.7%)	53 (88.3%)	OR 2.99 (1.14–7.88)		
	T3	41 (68.3%)	52 (86.7%)	OR 3.01 (1.20–7.57)		
Nerve block success rate	T1	41 (68.3%)	49 (81.7%)	OR 2.06 (0.88–4.83)	Wald χ^2^(2) = 0.08	0.960
	T2	40 (66.7%)	50 (83.3%)	OR 2.50 (1.05–5.94)		
	T3	39 (65.0%)	49 (81.7%)	OR 2.40 (1.03–5.57)		
Intubation time (s)	T1	65.1 (11.9)	58.9 (10.8)	MD −6.20 (−10.31 to −2.09)	*F*(2, 236) = 1.16	0.561
	T2	66.0 (12.3)	59.2 (10.7)	MD −6.80 (−10.97 to −2.63)		
	T3	66.8 (12.6)	60.1 (11.0)	MD −6.70 (−10.98 to −2.42)		
Errors per intubation	T1	1.8 (0.7)	1.2 (0.6)	MD −0.60 (−0.84 to −0.36)	*F*(2, 236) = 2.26	0.323
	T2	1.9 (0.8)	1.3 (0.6)	MD −0.60 (−0.86 to −0.34)		
	T3	2.0 (0.9)	1.4 (0.7)	MD −0.60 (−0.89 to −0.31)		
OSATS technical score (0–25)	T1	16.4 (2.8)	18.3 (2.7)	MD 1.90 (0.91–2.89)	*F*(2, 236) = 1.81	0.404
	T2	16.1 (2.9)	18.0 (2.6)	MD 1.90 (0.90–2.90)		
	T3	15.7 (3.0)	17.7 (2.7)	MD 2.00 (0.97–3.03)		

OR, odds ratio; MD, mean difference (VR–TBP minus control); OSATS, Objective Structured Assessment of Technical Skills. Binary outcomes (intubation success rate and nerve block success rate) were analyzed using generalized estimating equations (GEE) with a logit link; group × time interactions were tested using Wald χ^2^ statistics with 2 degrees of freedom. Continuous outcomes (intubation time, errors per intubation, OSATS technical score) were analyzed using linear mixed-effects models with fixed effects for group, time, and group × time and a random intercept for participants; interactions were tested using Type III F-tests with 2 and 236 degrees of freedom.

Ultrasound-guided nerve block success showed a similar advantage for VR-TBP, reaching statistical significance at T2 (83.3% vs. 66.7%, *p* = 0.038) and T3 (81.7% vs. 65.0%, *p* = 0.041). Technical competence assessed by OSATS was also consistently higher in the VR-TBP group (e.g., T3: 17.7 ± 2.7 vs. 15.7 ± 3.0, *p* = 0.003).

Overall, VR-TBP residents demonstrated superior and sustained technical proficiency, with benefits evident immediately after training and persisting through the 12-month follow-up.

### 3.3 Non-technical skills and team competence

**FIGURE 3:**
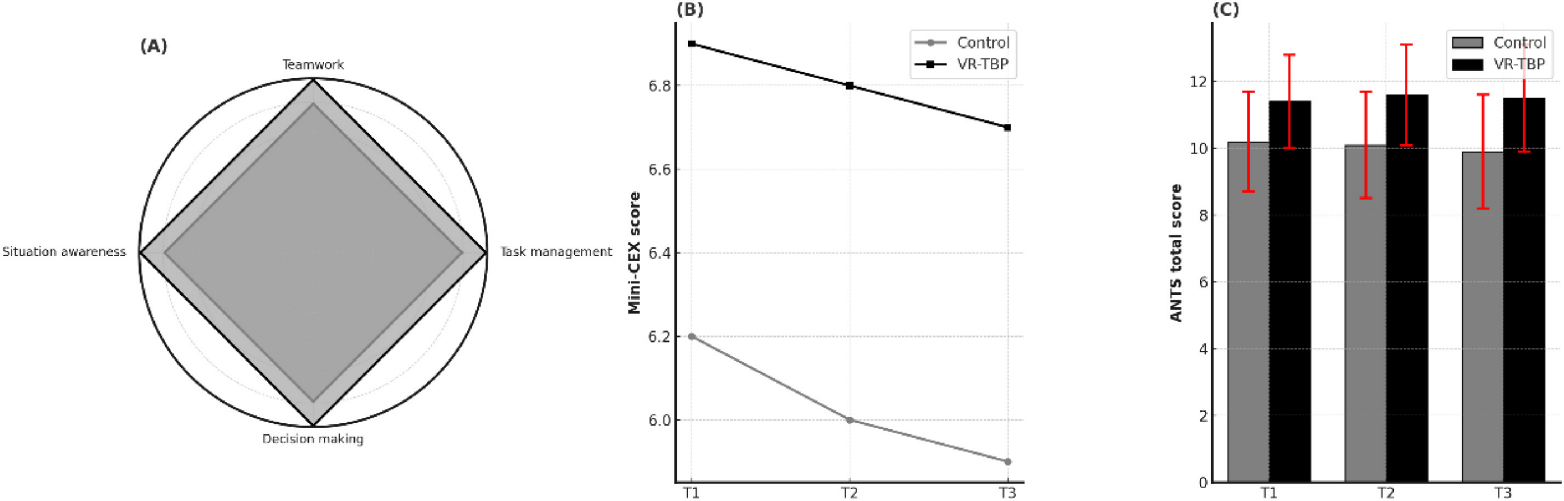
Non-technical skills and team competence outcomes. **(A)** Radar plot of ANTS sub-dimensions (task management, teamwork, situation awareness, and decision making) at T3, demonstrating consistently higher scores in the VR-TBP group compared with the conventional group. **(B)** Mini-CEX longitudinal scores across T1–T3, showing progressive improvement in clinical performance in both groups, with a greater increase in VR-TBP residents. **(C)** ANTS total scores (mean ± SD) at T1–T3, presented as bar graphs with error bars. The VR-TBP group achieved significantly higher scores than controls at all-time points (*p* < 0.001).

Non-technical performance consistently favored VR-TBP across all domains (Figure 3; Table 3). Mini-CEX scores were higher at every time point, with the difference sustained through 12 months (e.g., T3: 6.7 ± 1.0 vs. 5.9 ± 1.1, *p* < 0.001). Parallel improvements were observed in ANTS scores, where VR-TBP residents maintained a significant advantage from T1 onward (e.g., T3: 11.5 ± 1.6 vs. 9.9 ± 1.7, *p* < 0.001).

**TABLE 3:** Non-technical performance and secondary competency outcomes of anesthesiology residents at immediate post-intervention (T1), 6-month follow-up (T2), and 12-month follow-up (T3).

Indicator	Time point	Control mean (SD)	VR–TBP mean (SD)	Effect at time point, MD (95% CI)†	Group × time interaction, F (df)*	*p*-value (interaction)
ANTS non-technical skills (4–16)	T1	10.2 (1.5)	11.4 (1.4)	1.20 (0.68–1.72)	*F*(2, 236) = 1.42	0.244
	T2	10.1 (1.6)	11.6 (1.5)	1.50 (0.95–2.05)		
	T3	9.9 (1.7)	11.5 (1.6)	1.60 (1.01–2.19)		
Mini-CEX global score (1–9)	T1	6.2 (1.0)	6.9 (0.9)	0.70 (0.36–1.04)	*F*(2, 236) = 0.31	0.736
	T2	6.0 (1.1)	6.8 (1.0)	0.80 (0.42–1.18)		
	T3	5.9 (1.1)	6.7 (1.0)	0.80 (0.42–1.18)		
Theoretical exam score (0–100)	T1	75.0 (6.2)	78.3 (5.7)	3.30 (1.17–5.43)	*F*(2, 236) = 0.66	0.518
	T2	74.3 (6.5)	78.5 (6.1)	4.20 (1.94–6.46)		
	T3	73.8 (6.7)	78.1 (6.2)	4.30 (1.99–6.61)		
General Self-Efficacy Scale (10–40)	T1	28.7 (3.6)	30.4 (3.4)	1.70 (0.45–2.95)	*F*(2, 236) = 0.84	0.434
	T2	28.3 (3.7)	30.6 (3.5)	2.30 (1.01–3.59)		
	T3	27.9 (3.8)	30.2 (3.6)	2.30 (0.98–3.62)		

† MD, mean difference (VR–TBP minus control) with 95% confidence interval, derived from linear mixed-effects models adjusted for group, time, and group × time, with a random intercept for participants. *Group × time interaction tested using Type III F-tests from the mixed-effects models.

Theoretical knowledge also showed consistent benefits for VR-TBP, with higher exam scores across all assessments (T3: 78.1 ± 6.2 vs. 73.8 ± 6.7, *p* = 0.002). Similarly, self-efficacy remained superior in VR-TBP throughout follow-up (T3: 30.2 ± 3.6 vs. 27.9 ± 3.8, *p* = 0.004).

Together, these findings confirm that VR-TBP not only enhanced non-technical skills immediately after training but also sustained its advantages in teamwork, decision-making, and confidence up to 12 months.

### 3.4 Teaching feedback and learning experience

**FIGURE 4:**
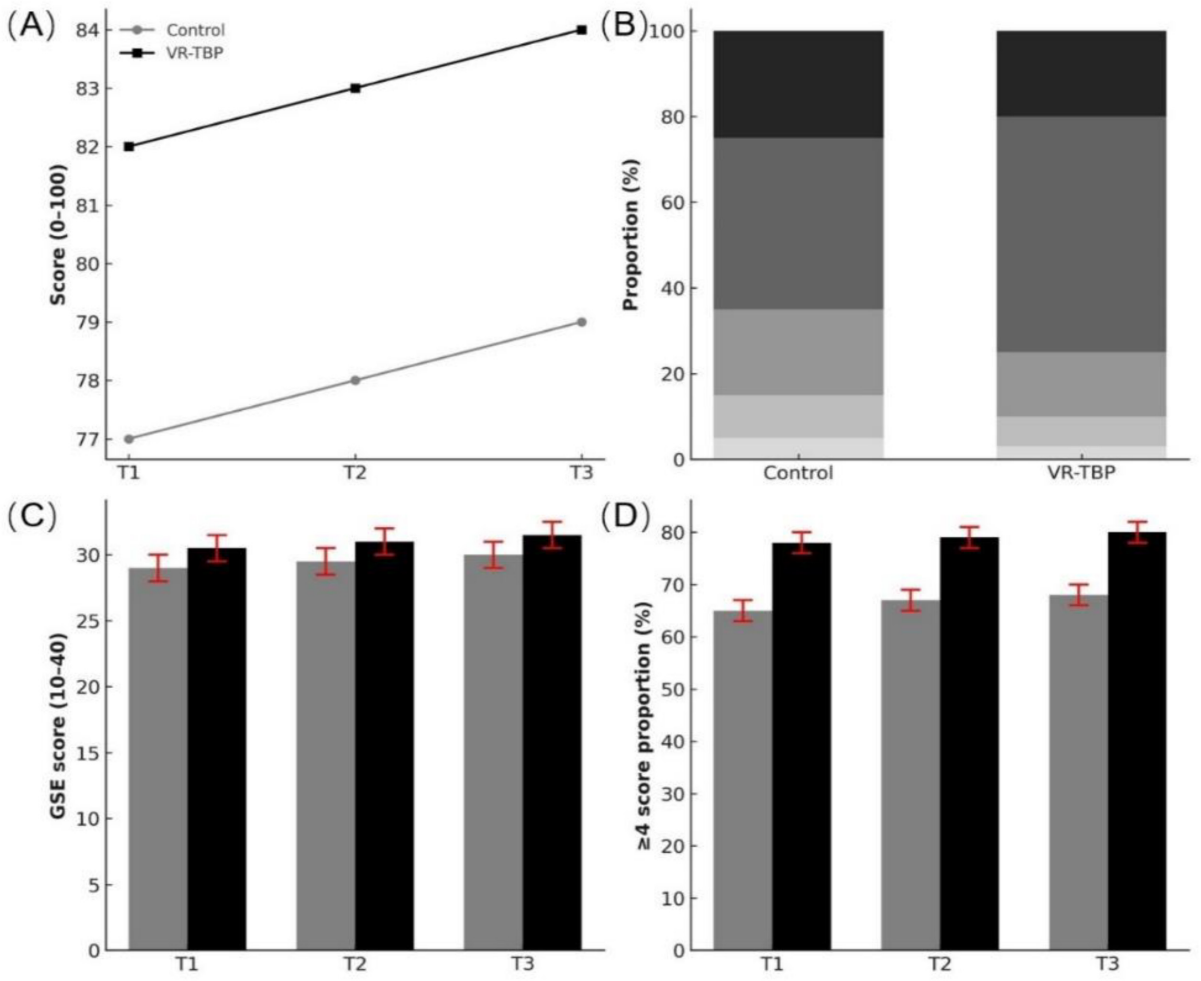
Teaching feedback and learning experience outcomes. **(A)** Mean theoretical examination scores (0–100) across T1–T3, showing consistently higher performance in the VR-TBP group. **(B)** Distribution of satisfaction scores on a 5-point Likert scale, with approximately 15% of participants in both groups rating “3” (moderate satisfaction). **(C)** General Self-Efficacy (GSE) scores (mean ± SD) at T1–T3; the VR-TBP group demonstrated significantly higher self-efficacy. **(D)** Proportion of participants rating satisfaction ≥ 4 across T1–T3, with consistently higher values in the VR-TBP group.

Teaching-related outcomes also favored VR-TBP (Figure 4; Table 3). Baseline theoretical knowledge and self-efficacy were comparable between groups. After training, VR-TBP residents achieved higher theoretical exam scores, with the advantage persisting through 12 months (T3: 78.1 ± 6.2 vs. 73.8 ± 6.7, *p* = 0.002). Similarly, self-efficacy increased more in VR-TBP from T1 onward and remained higher at T3 (30.2 ± 3.6 vs. 27.9 ± 3.8, *p* = 0.004).

Training satisfaction was high in both groups and showed no significant baseline difference (75.0% vs. 73.3%, *p* = 0.85), but consistently favored VR-TBP during follow-up. Overall, these findings indicate that VR-TBP enhanced not only objective knowledge acquisition but also residents’ confidence and learning experience.

### 3.5 Long-term follow-up outcomes

**FIGURE 5:**
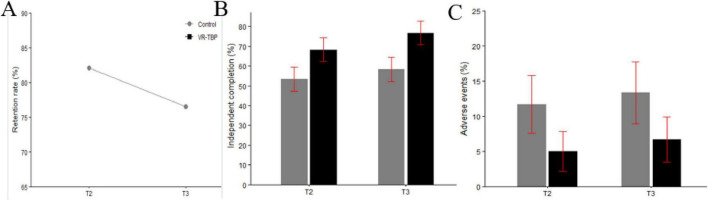
Long-term outcomes at 6-month (T2) and 12-month (T3) follow-up. **(A)** Skill retention (%) at T2 and T3, showing consistently higher values in the VR-TBP group compared with the Control group. **(B)** Proportion of independent procedure completion (%), with significantly greater independence observed in the VR-TBP group at T3. **(C)** Adverse event rates (%), indicating low complication frequencies in both groups with no statistically significant difference.

At the 6-month follow-up (T2), skill retention remained higher in the VR–TBP group than in controls (91.2 ± 8.7% vs. 82.1 ± 9.4%, *t* = −5.08, *p* < 0.001), and the proportion achieving independent clinical procedure completion was higher but did not reach statistical significance (68.3% vs. 53.3%, χ^2^ = 2.91, *p* = 0.088). At 12 months (T3), VR–TBP residents demonstrated superior skill retention (88.4 ± 9.5% vs. 76.5 ± 10.1%, *t* = −6.05, *p* < 0.001) and higher independent procedure completion (76.7% vs. 58.3%, χ^2^ = 4.62, *p* = 0.032). Adverse events were infrequent and did not differ significantly at either T2 (5.0% vs. 11.7%, *p* = 0.20) or T3 (6.7% vs. 13.3%, *p* = 0.24) Long-term outcomes are summarized in Figure 5 and Table 4.

**TABLE 4:** Long-term outcomes (skill retention, independent procedures, and adverse events) at 6-month (T2) and 12-month (T3) follow-up.

Outcome	Time point	Control group (Conventional, *n* = 60)	Intervention group (VR-TBP, *n* = 60)	Statistical test (value)	*P*-value
Skill retention (% of T1 performance), mean (SD)	T2	82.1 (9.4)	91.2 (8.7)	*t* = −5.08	< 0.001
	T3	76.5 (10.1)	88.4 (9.5)	*t* = −6.05	< 0.001
Independent clinical procedures, n (%)	T2	32 (53.3)	41 (68.3)	χ^2^ = 2.91	0.088
	T3	35 (58.3)	46 (76.7)	χ^2^ = 4.62	0.032
Adverse events, n (%)	T2	7 (11.7)	3 (5.0)	χ^2^ = 1.64	0.20
	T3	8 (13.3)	4 (6.7)	χ^2^ = 1.39	0.24

### 3.6 Subgroup analyses

**FIGURE 6:**
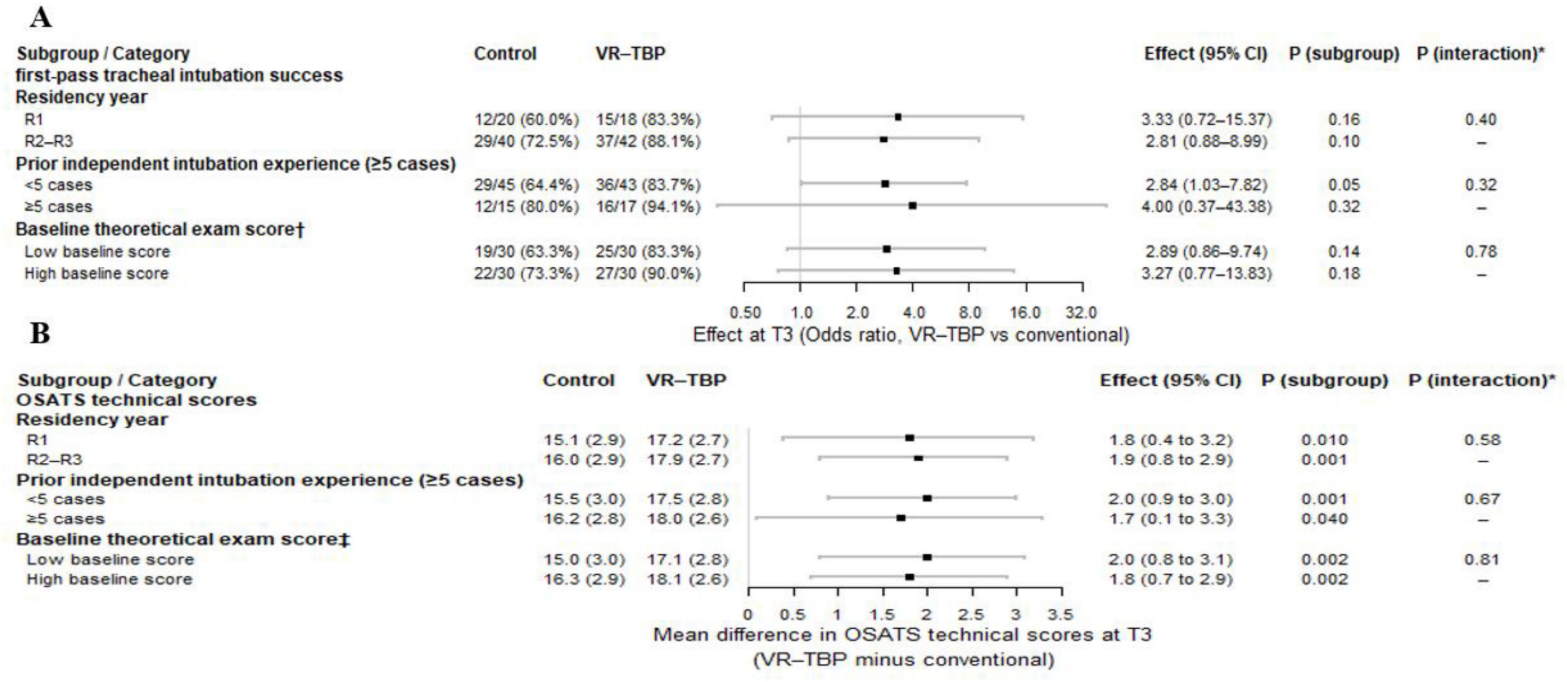
Subgroup analyses of **(A)** first-pass tracheal intubation success and **(B)** OSATS technical scores at T3 comparing VR-TBP with conventional training. Squares indicate odds ratios **(A)** or mean differences **(B)**; horizontal lines represent 95% confidence intervals. P (subgroup) is the P-value for the comparison between VR-TBP and control within each subgroup. P (interaction) is the P-value for the interaction between treatment group and the corresponding subgroup (test for heterogeneity of effect). Baseline theoretical exam score / : participants were categorized into low and high baseline score groups according to the median value of the baseline theoretical exam.

At T3, subgroup analyses based on GEE models indicated directionally consistent improvements in first pass tracheal intubation success with VR–TBP versus control across residency year, prior independent intubation experience, and baseline theoretical examination score strata. Success increased from 60.0 to 83.3% in R1 residents, with OR 3.33 and 95% CI 0.72–15.37(*p* > 0.05), and from 72.5 to 88.1% in R2–R3 residents, with OR 2.81 and 95% CI 0.88–8.99(*p* > 0.05). Among residents with fewer than 5 prior independent intubations, success increased from 64.4 to 83.7%, with OR 2.84 and 95% CI 1.03–7.82 (*p* < 0.05), whereas among those with at least 5 cases, success increased from 80.0 to 94.1%, with OR 4.00 and 95% CI 0.37–43.38 (*p* > 0.05). In the low and high baseline theoretical score subgroups, success increased from 63.3 to 83.3% with OR 2.89 and 95% CI 0.86–9.74 (*p* > 0.05), and from 73.3 to 90.0% with OR 3.27 and 95% CI 0.77–13.83 (*p* > 0.05), respectively. Group × subgroup interactions were not significant for residency year (Wald χ^2^ 0.72, df = 1, *p* > 0.05), prior experience (Wald χ^2^ 1.00, df = 1, *p* > 0.05), or baseline score (Wald χ^2^ 0.08, df = 1, *p* > 0.05), indicating no evidence of effect heterogeneity across the prespecified subgroups (Figure 6).

For OSATS technical scores at T3, VR–TBP was associated with higher mean scores within all subgroups. By residency year, scores increased from 15.1 ± 2.9 to 17.2 ± 2.7 in R1 residents, with a mean difference of 1.8 and 95% CI 0.4–3.2 (*p* < 0.05), and from 16.0 ± 2.9 to 17.9 ± 2.7 in R2–R3 residents, with a mean difference of 1.9 and 95% CI 0.8–2.9 (*p* < 0.05). By prior independent intubation experience, scores increased from 15.5 ± 3.0 to 17.5 ± 2.8 with a mean difference of 2.0 and 95% CI 0.9–3.0 (*p* < 0.05) in the fewer than 5 cases subgroup, and from 16.2 ± 2.8 to 18.0 ± 2.6 with a mean difference of 1.7 and 95% CI 0.1–3.3 (*p* < 0.05) in the at least 5 cases subgroup. By baseline theoretical score, scores increased from 15.0 ± 3.0 to 17.1 ± 2.8 with a mean difference of 2.0 and 95% CI 0.8–3.1 (*p* < 0.05) in the low score subgroup, and from 16.3 ± 2.9 to 18.1 ± 2.6 with a mean difference of 1.8 and 95% CI 0.7–2.9 (*p* < 0.05) in the high score subgroup. Interaction testing remained non-significant for residency year (Wald χ^2^ 0.31, df = 1, *p* > 0.05), prior experience (Wald χ^2^ 0.18, df = 1, *p* > 0.05), and baseline score (Wald χ^2^ 0.06, df = 1, *p* > 0.05), supporting consistent improvements in technical performance without detectable subgroup-dependent differences (Figure 6).

## 4 Discussion

This randomized controlled trial provides robust evidence that integrating VR–based immersive simulation with a structured team-based pedagogical framework (VR-TBP) yields consistent and durable improvements in anesthesiology resident training. Compared with conventional methods, VR-TBP was associated with higher first-pass intubation success, shorter intubation times, fewer procedural errors, and superior performance in ultrasound-guided nerve block. These technical advantages were paralleled by significant gains in non-technical domains, including higher Mini-CEX and ANTS scores, improved theoretical knowledge, greater self-efficacy, and enhanced training satisfaction. Importantly, these benefits persisted through 12 months, with superior skill retention and higher rates of independent procedure completion in the VR–TBP group. Adverse events were infrequent and did not differ significantly between groups. Together, these findings highlight VR-TBP as a comprehensive educational strategy that strengthens both immediate and long-term competence.

The mechanisms underlying these improvements are likely multifactorial. VR provided residents with a standardized yet immersive environment that enabled deliberate, repetitive practice of high-stakes procedures in the absence of patient risk. This aligns with a prior study showing that VR-based training can improve airway management efficiency and crisis response skills (14). However, unlike earlier studies which were often limited by brief interventions and narrow technical focus, the current trial integrates both technical and non-technical domains within a pedagogically grounded structure. By incorporating a structured team-based pedagogy, our trial addressed essential competencies such as communication, teamwork, and decision-making under pressure—skills previously shown to influence perioperative safety (15) but rarely targeted simultaneously in simulation-based research (16). The observed gains in ANTS and Mini-CEX scores reinforce findings that team-based immersive simulation enhances cognitive adaptability and behavioral coordination beyond what VR-only models can achieve (17).

From an educational perspective, these findings underscore the value of VR-TBP as a reproducible and scalable training model. Residency programs worldwide are challenged by variability in clinical case exposure, constraints on faculty supervision, and the need for competency-based progression. Our results suggest that VR-TBP can provide a standardized platform to ensure equitable opportunities for skill acquisition and retention across trainees. Moreover, the durability of benefits observed at 12 months is particularly relevant, as long-term consolidation of competencies has rarely been evaluated in previous VR studies (18).

Beyond anesthesiology, the implications of VR-TBP extend to other procedural specialties where technical proficiency and team-based coordination are critical, such as emergency medicine, critical care, and surgery. Prior VR studies in these domains have demonstrated localized benefits, but lacked integration into structured curricular frameworks (19, 20). By aligning immersive practice with collaborative pedagogy, VR-TBP may serve as a generalizable model for modernizing residency curricula across disciplines.

## 5 Limitations

Several limitations should be acknowledged. First, this was a single-center study conducted in a tertiary teaching hospital, which may limit the generalizability of the findings to residency programs with different structures or resources. Similar concerns about generalizability have been noted in previous VR training studies, where institutional factors influenced trainee engagement and curriculum fit (21).

Second, although randomization and allocation concealment were rigorously implemented, participants could not be blinded to the training modality. This is a common challenge in simulation-based education, where perceived novelty and performance bias may affect subjective measure such as satisfaction or self-efficacy (22).

Third, although Mini-CEX and ANTS assessments were performed by blinded faculty using standardized instruments, these tools inevitably involve some degree of subjectivity. Previous studies have similarly reported that faculty-rater variability can influence assessment outcomes in VR settings (23).

Fourth, the follow-up period was restricted to 12 months. Although this enabled evaluation of medium-term retention and clinical independence, longer-term effects extending into subsequent stages of professional practice remain unknown. This limitation is consistent with the broader VR education literature, where long-term durability of skill transfer is often insufficiently assessed (24).

Finally, adverse events were relatively infrequent in both groups, which limited statistical power to detect potential differences in safety outcomes. As prior pilot trials have also shown, low event frequency in controlled VR environments can restrict safety signal detection (25, 26).

## 6 Strengths

Despite these limitations, the study has several notable strengths. To our knowledge, it is among the first randomized controlled trials to evaluate a VR-integrated team-based pedagogical framework in anesthesiology residency while addressing both technical and non-technical domains.

The trial was prospectively powered, used rigorous randomization and allocation concealment, and applied blinded assessments to minimize bias. These elements directly address prior concerns in simulation-based education studies, where small sample sizes, inadequate randomization, and lack of assessor blinding were common methodological limitations.

All evaluation tools were validated Chinese versions, ensuring cultural appropriateness and comparability. This contrasts with many previous studies that directly applied Western-developed instruments without cross-cultural adaptation, raising concerns about validity in non-English-speaking populations.

The inclusion of assessments at baseline, immediately post-intervention, 6 months, and 12 months enabled evaluation of both short-term gains and medium-term retention. Previous VR-related research has rarely incorporated such longitudinal assessment frameworks, limiting the understanding of sustained skill consolidation.

Finally, the consistent superiority of VR-TBP across multiple domains—technical performance, non-technical skills, theoretical knowledge, self-efficacy, and satisfaction—underscores the robustness of the findings and highlights the potential of this approach for modernizing residency education.

## 7 Conclusion

This randomized controlled trial demonstrates that integrating VR-based simulation with multilevel team-based pedagogy enhances both technical and non-technical competencies in anesthesiology residents, with benefits persisting up to 12 months. These findings support the adaptation of this model to other procedure-oriented specialties.

## Data Availability

The raw data supporting the conclusions of this article will be made available by the authors, without undue reservation.
